# Green Synthesis of Curcuminoids-Enriched Trimetallic Nanoparticles Stabilised by Chitosan/Collagen Nanocomposites against Foodborne Pathogens

**DOI:** 10.1007/s12010-025-05565-x

**Published:** 2026-02-26

**Authors:** Mahmoud Emam, Mohamed Seif, Mohamed S. Hasanin

**Affiliations:** 1 Phytochemistry and Plant Systematics Department, National Research Centre, Dokki, 12622 Giza Egypt; 2 Food Toxicology and Contaminants Department, Food Industries and Nutrition Research Institute, National Research Centre, Dokki, 12622 Giza Egypt; 3 Cellulose& Paper Department, National Research Centre, El-Buhouth St, Dokki, 12622 Egypt

**Keywords:** Antimicrobial, Curcuminoids, Cytocompatibility, Foodborne pathogens, Nanocomposites, Trimetallic nanoparticle, Turmeric, Biopolymers

## Abstract

The integration of natural bioactive components with multiple metal nanoparticles in bionanocomposites will enhance their biological activities. In this study, a curcuminoid-rich extract was used as a capping and reducing agent for trimetallic nanoparticles (Ag@TiO_2_@ZnO), which were stabilized using biopolymers (chitosan and collagen). The formulated nanocomposite-based trimetallic nanoparticles were prepared using different ratios of curcuminoid extract (1%, 3%, and 5% *w/w*), labelled as CR-1, CR-2, and CR-3, respectively. Their physicochemical properties were characterized using UV-vis, FTIR and DLS. The morphological structure was characterized using FE-SEM with EDX, HR-TEM, and SAED. Additionally, their potential antimicrobial activities were evaluated against various strains of selected foodborne pathogenic bacteria and fungi. The physicochemical characterization confirmed that the curcumin ratio influenced the size and shape of the trimetallic nanoparticles, as well as the network structure of the stabilized biopolymer matrix. Among the formulations, CR-2 exhibited the most potent activity against all tested foodborne microorganisms of pathogenic bacterial strains with MIC ranges of 0.4 to 0.9 mg/mL and mycotoxigenic fungal strains with MIC of 0.2 to 1.1 mg/mL, along with excellent cytocompatibility with human skin fibroblasts (BJ-1), showing a cell viability of 96.17%. The nanoscale size of CR-2 was recorded between 52 and 11 nm. The average zeta potential was recorded as 23 ± 4 mV, indicating high colloidal stability. The findings highlight the high potential of these green-synthesized trimetallic nanoparticles, particularly CR-2, for commercial development in safe therapeutic and cosmetic formulations, as well as potential applications in agriculture and food safety.

## Introduction

Food safety is a crucial issue for public health. Unsafe food leads to millions of illnesses and deaths worldwide each year. Adhering to proper food hygiene practices can significantly lower this burden of disease [1]. Foodborne pathogens, including bacteria, viruses, and parasites, pose a significant threat to global health, leading to foodborne illnesses, particularly in regions with inadequate food safety regulations [2–4]. These microbes are extremely resilient, able to thrive in diverse environmental conditions and often impervious to conventional food processing methods. Although the widespread use of synthetic antimicrobial agents in food preservation has reduced their occurrence, continuous exposure has also led to a concerning upsurge in their antimicrobial resistance [5, 6]. Moreover, synthetic antimicrobial agents pose many health hazards for humans and the environment due to their residues in food [7–10]. For these concerns, there is a growing interest in exploring natural antimicrobial agents as alternatives to chemical-based ones. One approach involves the use of natural compounds, such as phytochemicals, which possess antioxidant and antimicrobial properties [11, 12].

The use of natural antimicrobial agents as food preservatives shows promise in food preservation, but still faces challenges that consequently limit their use, such as poor solubility, low bioavailability, and rapid degradation [13]. To overcome these limitations, advances in biotechnology approaches, especially the formulation of nanocomposites based on tri-metallic compounds through green synthesis, present a promising solution.

By combining metals such as silver [14], zinc oxide [15, 16], titanium oxide [14, 17], and copper oxide [18], these nanocomposites enhance antimicrobial efficacy while simultaneously reducing toxicity and environmental impact [19–21]. Trimetallic nanoparticles have garnered considerable attention among the various categories of nanoparticles [22]. Nanotechnology and nanoparticles have a strong potential for product and process innovation in the food industry sector [23]. By combining three separate metals, trimetallic nanoparticles can enhance the unique features of each metal, surpassing the performance of monometallic or bimetallic counterparts [24]. The integration of multiple metals enables the tuning of properties such as catalytic activity, electrical conductivity, and optical activity [25]. Metal and metal oxide nanoparticles are widely used in the biomedical and pharmaceutical sectors. Among these metal oxides, titanium dioxide (TiO₂) is known for its versatile properties, making it suitable for a wide range of biological applications [26]. Likewise, zinc oxide (ZnO) nanoparticles have also gained attention for their unique antibacterial, antifungal, and UV-filtering capabilities [27]. Similarly, silver nanoparticles (AgNPs) are highly effective against bacterial, fungal, and viral infections and exhibit low toxicity to humans at minimal concentrations, making them a valuable addition to antibiotics in addressing the issue of synthetic antimicrobial resistance [28].

Trimetallic nanoparticles (TMNPs) exhibit superior physicochemical and catalytic properties compared to their mono- and bimetallic counterparts due to the synergistic interactions among the three constituent metals [29]. These interactions enhance electronic, optical, magnetic, and catalytic behaviors, resulting in higher activity, selectivity, and stability across a wide range of applications, including catalysis, sensing, antimicrobial action, and environmental remediation [19]. The combination of three metals enables TMNPs to possess multiple active sites and optimized surface energies, resulting in improved reaction kinetics and durability under operational conditions. Furthermore, trimetallic systems often demonstrate enhanced electrochemical and photocatalytic efficiency, making them more effective in pollutant degradation and energy conversion than mono- or bimetallic nanoparticles [29]. Overall, TMNPs represent a new generation of multifunctional materials with enhanced reactivity, improved performance, and increased environmental sustainability [30].

Biopolymers such as chitosan and collagen are commonly used as stabilizers for metal nanoparticles due to their biocompatibility and positive charge [31]. Chitosan, in particular, is known for its effectiveness in stabilising metal nanoparticles. Collagen, on the other hand, is non-toxic and has minimal antigenicity, making it suitable for various biomedical and nutritional applications [32, 33].

Complementing the enhancement of biopolymers efficiency, bioactive natural materials use, *Curcuma longa* L., commonly known as turmeric or “golden spice,” is a flowering plant belonging to the family Zingiberaceae. Since ancient times, turmeric has been widely recognized for its versatile applications as a medicinal ingredient, a food additive, and in the field of nutraceuticals. Additionally, it has been explored for its development of various impressive antioxidant and antimicrobial properties, indicating its utility in the food, nutraceutical, cosmetic, and pharmaceutical industries [34, 35].

The diverse activities and commercial benefits of turmeric are attributed to its chemical composition and metabolites, including minerals (3.5%), carbohydrates (69.4%), fat (5.1%), protein (6.3%), and moisture (13.1%). The oil obtained by steam distillation of the rhizomes contains α-phellandrene (1%), cineol (1%), borneol (0.5%), sapine (0.6%), zingiberene (25%), and sesquiterpene (53%). In addition to curcumin, which makes up 96% of the chemical curcumin (3–4%), is responsible for its yellow color [36]. In light of these attributes, the present study was designed to formulate nanocomposites based on curcuminoid-rich extract to enhance its biological properties. The curcuminoids-rich extract was used at various ratios (1, 3, and 5% *w/w*) as capping and reducing agents, stabilized with high-bioactive polymers, chitosan and collagen. The synthesized trimetallic nanocomposites were prepared using green methods. The physicochemical and ultrastructural properties of the formulated nanocomposites were examined. Furthermore, their antimicrobial activities were evaluated against foodborne bacterial and fungal pathogens, while also assessing their cytocompatibility with normal human skin fibroblasts (BJ-1) cells.

## Materials and methods

### Materials

HPLC-grade solvents were used for the HPLC analysis. Every additional chemical utilised in the existing extraction process was of analytical grade. Zn acetate pentahydrate was purchased from Loba Chem India. Titanium tetrachloride and silver nitrate were purchased from Sigma Aldrich, Germany. Low-molecular-weight chitosan and Bovine collagen type I were purchased from Sigma-Aldrich USA. Microbiological media were purchased from Sigma Aldrich Spain. All chemicals, reagents, and culture media were used as received.

### Plant Collection and Extract Preparation

#### Rhizomes of *Curcuma Longa* L. (turmeric) (CR) and Extract Preparations

The Rhizomes turmeric (*Curcuma longa* L.) was obtained from the Egyptian market in May 2023, the underground rhizomes were cleaned, dried in the shade (at room temperature), crushed into powder (200 g), macerated with solvent (50% ethanol), and extracted using a sonication bath for 30 min four times per day, and defatted using *n*-hexane. The filtrates were dried using a Rotavapor^®^ (Heidolph, Schwabach, Germany), and the yield weight was recorded as ~ 63 g of powder extract. The lyophilized extract (Christ, Osterode am Harz, Germany) was stored at −20 °C.

### Phytochemical Analysis

#### HPLC Conditions

**Fig. 1:**
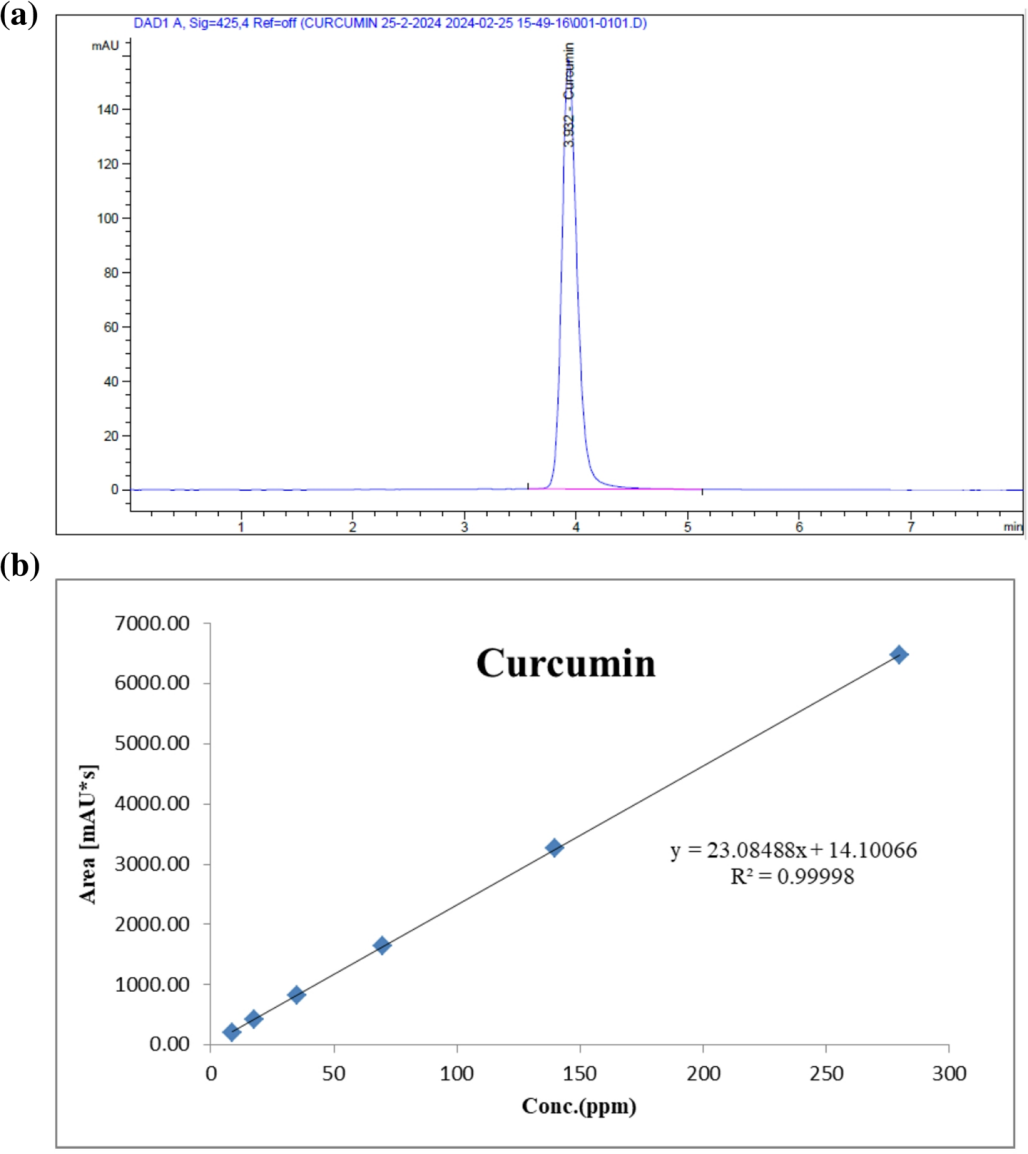
Curcumin standard retention time using HPLC (**a**) and standard curve of curcumin using HPLC (**b**)

**Fig. 2:**
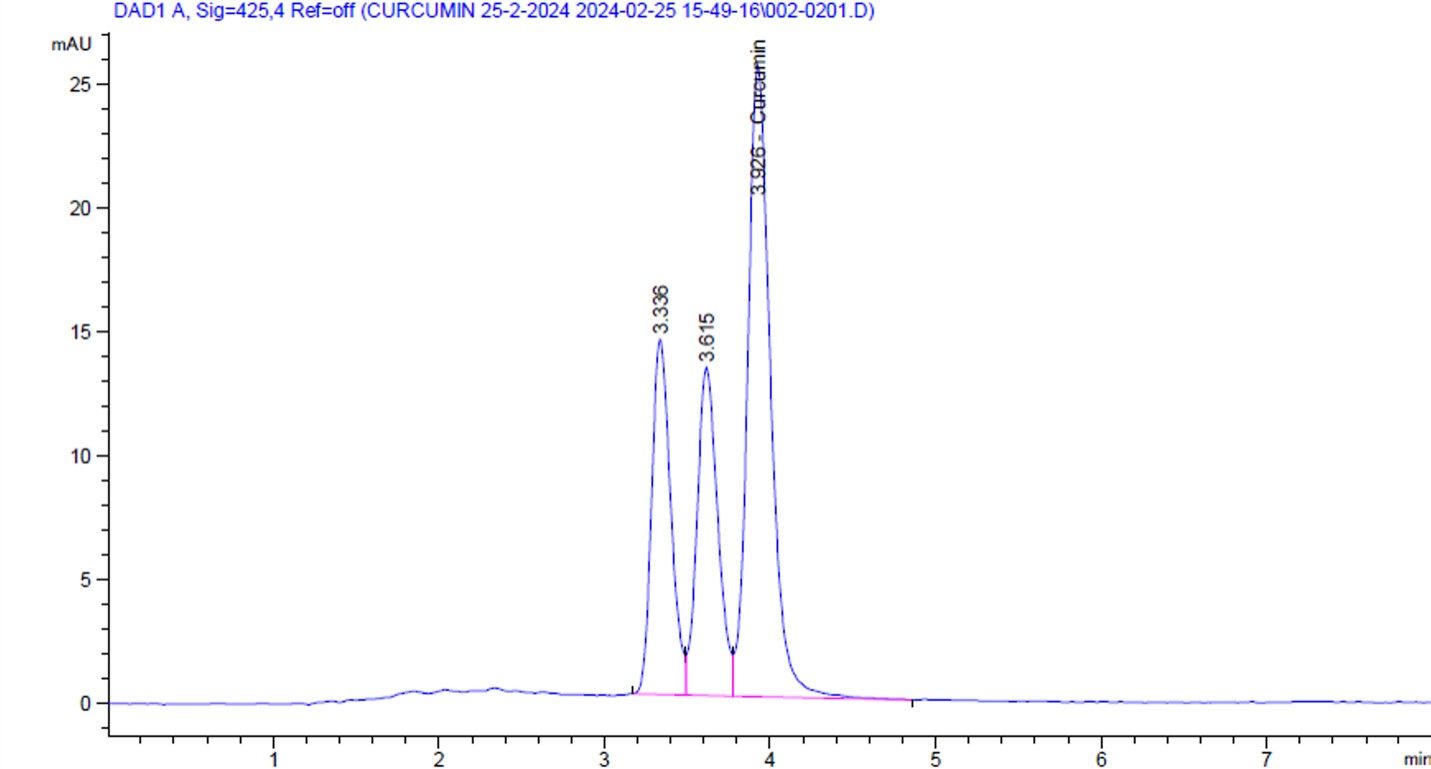
Curcuminoids**-**rich extract (Turmeric extract) chemical profile using HPLC

The curcuminoid-rich extract of turmeric was described for its content using HPLC. HPLC analysis was carried out using an Agilent 1260 series. For Curcumin, the column used was Agilent C18 (4.6 mm x250 mm i.d., 5 μm). The mobile phase was ACN: 2% acetic acid (50:50, *v/v*), and the flow rate was 2 mL/min. The injection volume was 5 µl for each of the sample solutions. The MWD was adjusted at 425 nm. The column temperature was maintained at 40 °C. The standard curcumin sample (Fig. 1a) was used to obtain the standard curve (Fig. 1b), which was carried out using HPLC, and the concentration of curcuminoid structures (Fig. 2) was evaluated using the standard curve.

#### LC-ESI-MS/MS Analysis

The turmeric extract was examined to confirm the presence of the main peaks in the sample using liquid chromatography–electrospray ionization–tandem mass spectrometry (LC-ESI-MS/MS) (AB Sciex ExionLC AC HPLC coupled with a SCIEX Triple Quad™ 5500 LC-MS/MS system) as described previously [37, 38].

### Nanocomposites Formulation

The nanocomposites were formulated using a green method that involved curcuminoids as both a reducing agent and capping agent, and chitosan as a stabilizing and distribution matrix, supported by collagen to enhance cytocompatibility [39]. Metal salts were used as a source of nanoparticles, namely, AgNO_3_, Zinc acetate hydrate, and Titanium tetrachloride. Herein, chitosan was dissolved in 1% acetic acid, and collagen was added at a concentration of 10% (*w/w*) based on the chitosan weight. This solution was stirred at 70 °C for 2 h until complete dissolution. A curcuminoid-rich extract was added in three ratios (1%, 3%, and 5% *w/w*) individually based on chitosan and continuously stirred for 3 h. Metal salts were added in equal weight (0.01 g) to the previously prepared solution and were continuously stirred overnight. The samples were named as CR-1, CR-2, and CR-3, respectively. White brownish color refers to the formulation of nanoparticles. The solution was ultrasonicated in the water bath for 3 h at 70 °C. The collected nanocomposites were harvested using absolute alcohol, washed three times with 70% alcohol, and centrifuged at 10,000 rpm for 15 min. The collected nanocomposites were lyophilized and preserved in a refrigerator for further use.

#### Characterizations

UV-Vis spectra were recorded on a Shimadzu model UV-240 spectrophotometer (JASCO, V-730, Japan) in the range of 200–800 nm. FTIR spectra were obtained using a Nicolet Impact-400 FT-IR spectrophotometer in the 400–4000 cm^− 1^ range. A morphological study was conducted using a field-emission scanning electron microscope (FE-SEM) Model Quanta 250 FEG, attached to an energy-dispersive X-ray (EDX) unit (FEI IN SPECTS Company, Philips, Poland). High-resolution transmission electron microscopy (HR-TEM) was used, specifically a Model JEM2010 from Japan, equipped with Selected Area Electron Diffraction (SAED). The average particle size distribution in (nm) and average zeta potential in (mV) were determined using a dynamic light scattering (DLS) equipment (Santa Barbara, CA, USA) under 23 °C conditions. The incoming light was the 632.8 nm line of a HeNe laser at an angle of 13.9°. The measurements were performed in triplicate, and the results were recorded as the mean with standard deviation values.

### Biological Evaluations

#### BJ-1 Cell Viability in Response To Exposure To the Formulated Nanocomposites

The viability of the normal human skin cell line (BJ-1 was evaluated under exposure to the formulated trimetallic nanocomposites. The cell was maintained in DMEM F12 medium supplemented with 10% fetal bovine serum, then incubated at 37 °C in 5% CO_2_ and 95% humidity. Sub-culturing of cells was performed using 0.15% trypsin. Cell viability was assessed using the MTT assay [40]. Briefly, cells were plated at a density of 10,000 cells per well in 96-well plates. After 24 h, the medium was replaced with serum-free medium containing the tested formulated composites at a final concentration of 100 µg/mL. The cells were treated in triplicate for 48 h [41].

#### Antimicrobial Activity of the Formulated Nanocomposites

##### Tested Microorganisms

The growth inhibitory influence of formulated nanocomposites was tested on six strains of foodborne pathogenic bacteria (Table 1). The bacterial cultures were grown on nutrient agar slants at 37 °C for 24 h and then stored in the refrigerator until used for the experiments.

**Table 1:** List of the pathogenic bacterial strains used in the study

Gram-positive bacterial strains		Gram-negative bacterial strains	
Bacterial strains	Identification code	Bacterial strains	Identification code
*Bacillus cereus*	EMCC 1080	*Escherichia coli*	0157 H7 ATCC 51,659
*Staphylococcus aureus*	ATCC 13,565	*Salmonella typhi*	ATCC 25,566
		*Pseudomonas aeruginosa*	NRRL B-272
		*Listeria monocytogenes*	LMD 7726

Similarly, Table 2 lists the six fungal species that were used in the antifungal test. After 5 days of growth at 25 °C on potato dextrose agar (PDA) slants, the fungal stock cultures were stored in the refrigerator until they were used.

**Table 2:** The fungal species used in the study

Fungal species	Identification code
*Aspergillus flavus*	NRRL 3357
*A. niger*	SSWT 2999
*A. ochraceus*	ITAL 14
*Fusarium verticillioides*	ITEM 10,027
*F. proliferatium*	MPVP 328
*Penicillium verrucosum*	BFE 500

##### Disc Diffusion Technique

From the 24-hour incubated nutrient agar slant of each bacterial species, a loopful of the microorganism was inoculated into tryptic soy broth. The bacterial cultures were uniformly spread from the tryptic soy broth using cotton swabs on the nutrient agar. The sterilized filter paper discs (6 mm) were loaded with the tested samples, 200 µL of 1 mg/mL of sample stock solution, and placed on the seeded plates using sterile forceps. DMSO represented the negative control, and tetracycline (500 µg/mL) was used as the positive control. After that, the inoculated plates were incubated at 37 °C for 24 h. At the end of the incubation period, inhibition zones were measured and expressed as the diameter of the clear zone, including the diameter of the paper disc. The fungal strains were plated onto PDA and incubated at 25 °C for 5 days. The inoculated plates were incubated at 25 °C for 24–48 h. At the end of the period, antifungal activity was evaluated by measuring the zone of inhibition (mm) against the tested fungus and a control positive, consisting of 1.0 mg/mL of miconazole (Sigma-Aldrich) [42]. All treatments consisted of three replicates, and the averages of the experimental results were determined.

#### Determination of MIC

The determination of MIC for formulated nanocomposites was conducted using the tube dilution method [43]. Culture tubes containing different concentrations of each sample, with ten serial dilutions starting at 10 mg/mL, were prepared. Each tube was inoculated with 100 µL of bacterial cell suspension and incubated at 37 °C for 24 h. Furthermore, MIC against fungi was determined using the technique described by Sokmen et al. (2004) with the same serial dilutions of the tested samples [44]. The plates were incubated at 25 °C for 24 to 72 h. At the end of the incubation period, mycelial growth was monitored, and the MIC was determined.

### Statistical Analysis

All data were collected from three replicates (*n* = 3) and are expressed as mean ± SE. In addition, GraphPad Prism 8.0.2 software was used to visualize the biological results.

## Results and Discussion

### Phytochemical Study

The prepared curcuminoids-rich extract from turmeric extract was yielded a (~ 63 g powder extract). The curcumin structure was determined using HPLC against its standard materials. Specifically, the results showed that the curcumin structure was present at a concentration of 22.59 mg/g in the powder extract. Moreover, the relative area percentage of the HPLC results (Fig. 2) indicated that the curcumin structure accounted for 51.51%. In addition, the peaks appeared at retention times of 3.3 min and 3.6 min, corresponding to concentrations of 24.11% and 24.37%, respectively. Subsequently, the following applications were used to determine the chemical structures of these different peaks.

**Fig. 3:**
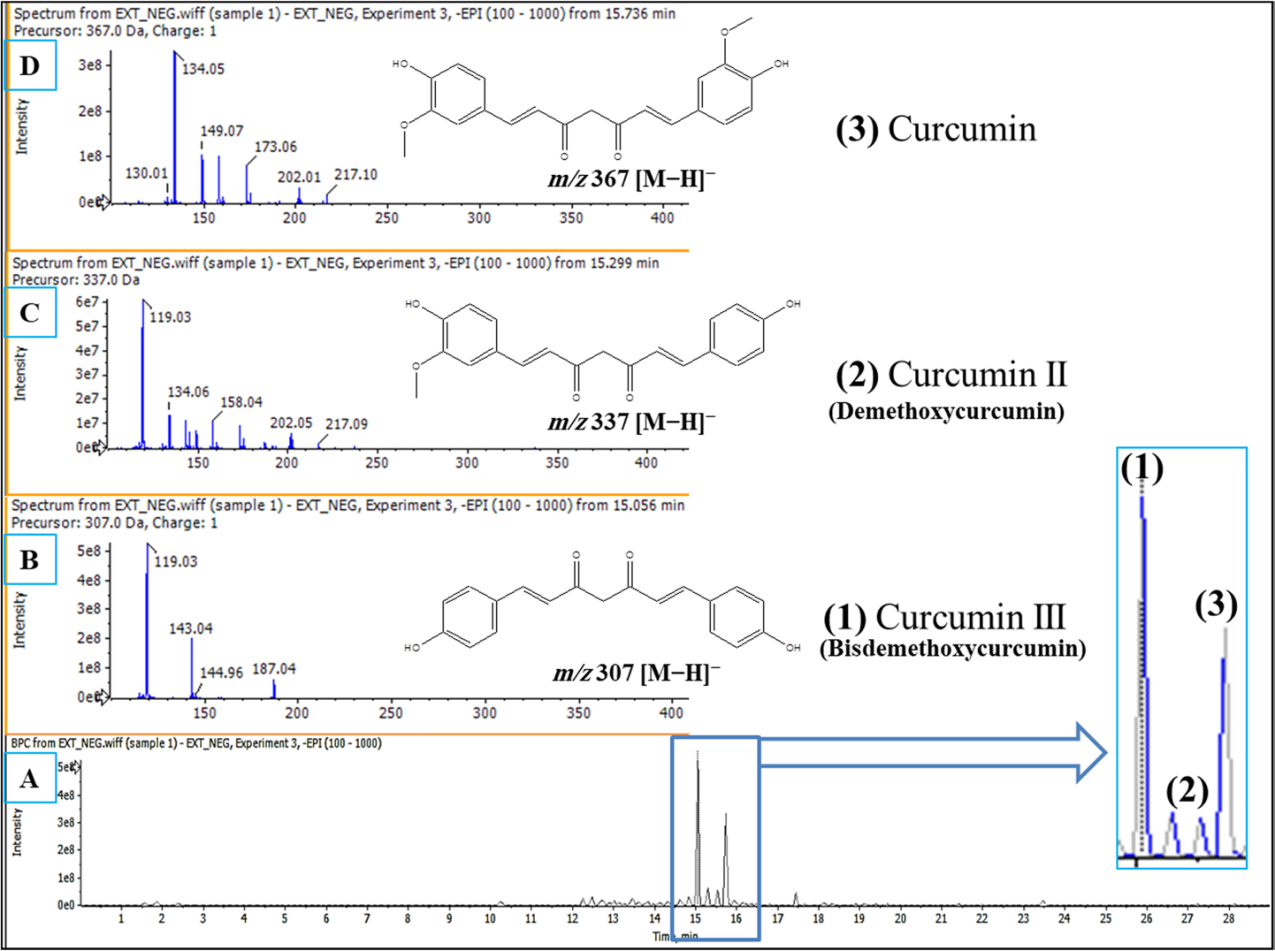
Base peak chromatogram (BPC) of curcuminoids-rich extract of Turmeric extract using LC-ESI-MS/MS [**A]**. Negative ion chromatograms of bisdemethoxycurcumin [**B]**, demethoxycurcumin [**C]**, and curcumin [**D]**, respectively

Furthermore, plant extracts can be effectively separated and identified using hyphenated liquid and gas chromatography in conjunction with mass detectors. One of its primary benefits is its high sensitivity and specificity, making it applicable to both volatile and non-volatile substances [45]. In this study, LC-ESI-MS/MS was employed to identify the major bioactive secondary metabolites in the curcuminoids-rich extract. This technique was used to tentatively identify the major secondary metabolites of turmeric extract as bisdemethoxycurcumin (*m/z* 307 [M-H]^−^), demethoxycurcumin (*m/z* 337 [M − H]^−^), and curcumin (*m/z* 367 [M − H]^−^) (Fig. 3). This identification was based on the analysis of the fragmentation ions (MS/MS) and a comparison with previously reported data [46].

### Nanocomposites Characterizations

**Fig. 4:**
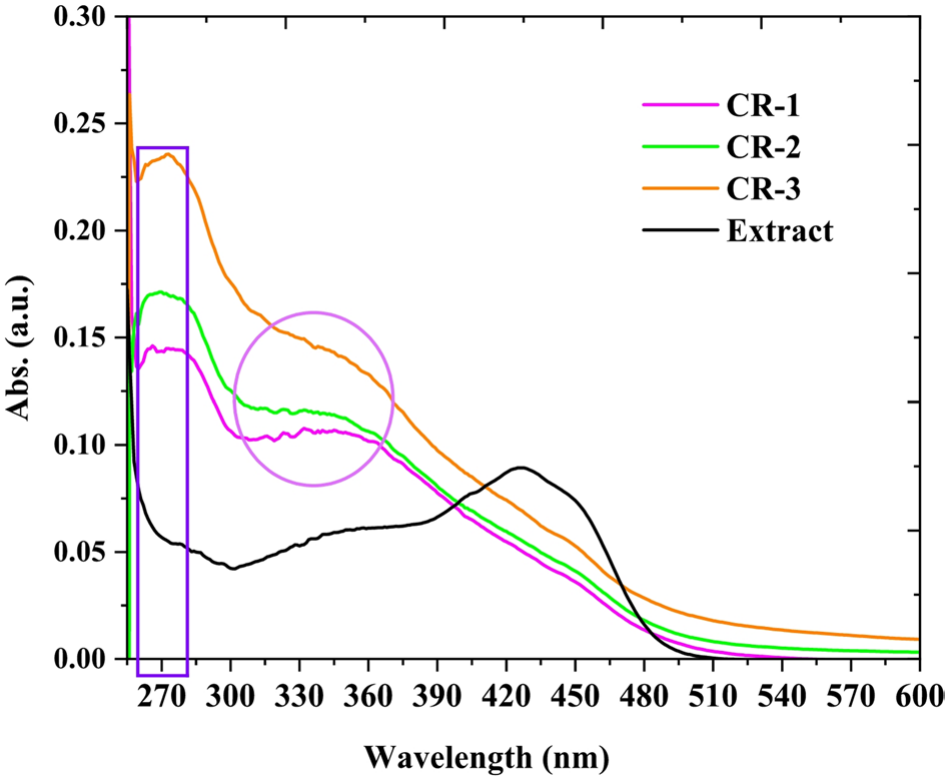
UV-vis of curcuminoids-rich extract and formulated nanocomposites

Nanocomposite formulas were prepared using a green biotechnology approach. In this process, curcuminoid-rich extract was applied in different ratios as a reducing and capping agent together with chitosan and collagen as stabilizing agents. Spectrochemical studies, including UV-vis, FTIR, and DLS, were employed to assess the physicochemical characterization of the formulated nanocomposites. As shown in Fig. 4, the UV-vis spectra for curcuminoid-rich extract and formulated nanocomposites were analyzed. A curcuminoid-rich extract spectrum exhibited a broad peak at 427 nm, which corresponded to the curcumin molecules, as reported in [47]. Moreover, all nanocomposites displayed a broad absorption band at 273 nm. Interestingly, small bands at 324, 331, and 363 nm were assigned in CR-1 and CR-2, but were absent in CR-3. This observation suggests that the trimetallic nanoparticles were successfully capped and exhibited shifted bands, which represented ZnONPs [48], AgNPs [49], and TiO_2_NPs [48] in CR-1 and CR-2. In contrast, these bands disappeared in CR-3, suggesting a distinct composition or behavior of nanoparticles. The conclusion was that the formulation of trimetallic nanoparticles was carried out via a curcuminoids-rich extract and involved in the biopolymer network, which is why the peak of the nanoparticles was shifted; these phenomena were in agreement with previous findings [37].

**Fig. 5:**
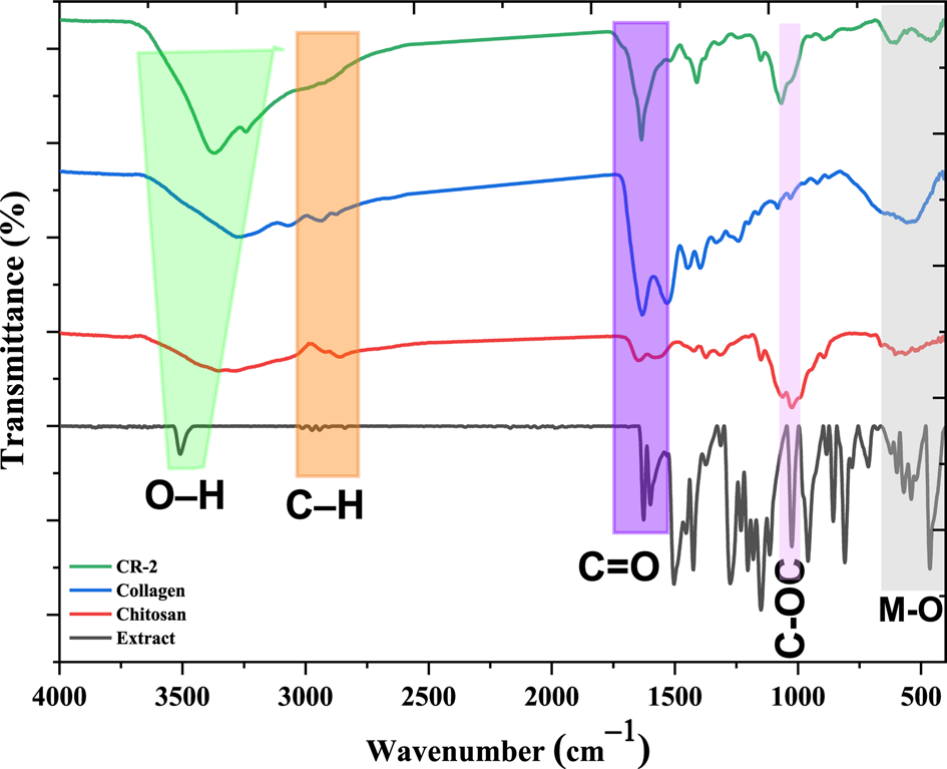
The FTIR spectra of curcuminoids-rich extract, pure chitosan, and collagen compared to nanocomposite (CR-2)

Furthermore, the FTIR spectra findings are presented in Fig. 5, showing a comprehensive range of vibrational motions that closely match the chemical skeletons of curcuminoids-rich extracts, specifically their aromatic rings, conjugated *β*-diketone moiety, methoxy substituents, and phenolic hydroxyl groups. The O–H stretching vibrations of phenolic hydroxyl groups are responsible for the broad and strong absorption band that usually appears between 3508 cm¹. Hydrogen bonds, which are prevalent in the solid state or in polar environments, often cause this band to broaden [47, 50]. While the stretching vibrations of CH3 and CH2 occurred at 2969 and 2921 cm^− 1^, respectively. Where aromatic C = C stretching bands may overlap or interact with this peak (1628) cm-¹. Benzene ring stretching vibrations appear at a peak of 1597 cm^− 1^. The C = O stretching vibration of the conjugated β-diketone system displayed a strong absorption band in the (1740 − 1680) cm⁻¹. Between 1500 and 1510 cm^–1^, additional aromatic skeleton vibrations that reflect symmetric C = C ring vibrations are detectable [47, 51]. Bending vibrations of olefinic C-H are recorded in the 1430 cm⁻¹ range, but weaker C–C stretching from the phenol structure in the 1420–1400 cm⁻¹ regions. Methoxy groups (-OCH₃) that are characteristic of curcuminoids, which result in weaker symmetric stretches between 1110 and 1070 cm^–1^ and strong asymmetric C–O–C stretching bands between 1240 and 1220 cm^–1^ [52]. C–O stretching vibrations are typically observed around 1300 to 1000 cm^–1^, and in-plane C–H bending vibrations from the aromatic rings appear in the 1000–650 cm^–1^ region. Further down in the spectrum, out-of-plane deformations of olefinic and aromatic C–H bonds appear between 1000 and 600 cm^–1^ [53, 54]. Chitosan spectrum exhibited prominent peaks at 577 cm^–1^ (out-of-plane bending NH, out-of-plane bending C–O), 1021 cm^–1^ (C–O–C stretching), 2866 cm^–1^ (CH_2_ stretching), and 3335 cm^–1^ (− OH stretching). Vibrational mode of amide C═O stretching was detected at 1644 and 1569 cm^–1^ [55]. Collagen spectrum observed bands at 3275 and 3006 cm^–1^ corresponding to amide A and OH, and amide B, respectively. The bands of CH stretching vibration were assigned at 2939 and 2881 cm^–1^. 1628, 1531, 1247 cm^–1^ were attributed to amide I, amide II, and amide III, respectively. The carbohydrate band was assigned at 1080 cm^–1^ [56]. On the other hand, the CR-2 nanocomposite spectrum illustrated the overlapping NH and OH bands at 3369 cm–1, as well as a small band at 3240 cm–1, which could be attributed to the amide A band of collagen. Additionally, the band at 1635 cm^–1^ was assigned as a high-intensity, sharp band. The carbohydrate band appeared at 1070 cm^–1^. These observations were confirmed by the change in the molecular structure of the nanocomposite following the integration of the components. Moreover, the fingerprint bands were assigned at 614, 556, and 460 cm^–1^, which enhanced the presence of a trimetallic structure within the nanocomposite.

DLS measurements in Table 3 calculated that the particle size of CR-1 was detected as 146 ± 14 nm with heterogeneous size due to PDI value (0.6 ± 0.1) [57]. Moreover, the average zeta potential was recorded as −9 ± 3 mV, which referred to a low stability colloidal solution [58]. An increase in curcuminoids-rich extract (CR-2) was performed, resulting in a decrease in nanocomposite particle size (76 ± 9 nm) with the low PDI value, which refers to the small nanoparticle formulation, and average zeta potential was recorded as −23 ± 4 mV, also recorded as having the highest stability behaviour compared with the above formulations. In addition, the increase in curcuminoids-rich extract particle size was decreased to 114 ± 12 nm, with a PDI of 0.6 ± 0.1, indicating moderate particle size homogeneity. The average zeta potential was recorded as −15 ± 4 mV, which referred to moderate colloidal stability, which is in agreement with the particles behavior and size. These studies concluded that the ratio of curcuminoid-rich extract controls the reduction of trimetallic to nanosized. Moreover, increasing the ratio of curcuminoids-rich extract to 5% acts as a feedback mechanism, resulting in a high rate of accumulation of trimetallic nanoparticles within the biopolymer network, accompanied by an increase in size and a decrease in stability compared to 3% nanocomposites.

**Table 3:** DLS measurements of formulated nanocomposites

Samples	Particles (nm)	PDI	Zeta potential (mV)
CR-1	146 ± 14	0.6 ± 0.1	−9 ± 3
CR-2	76 ± 9	0.3 ± 0.1	−23 ± 4
CR-3	134 ± 12	0.4 ± 0.1	−15 ± 4

**Fig. 6:**
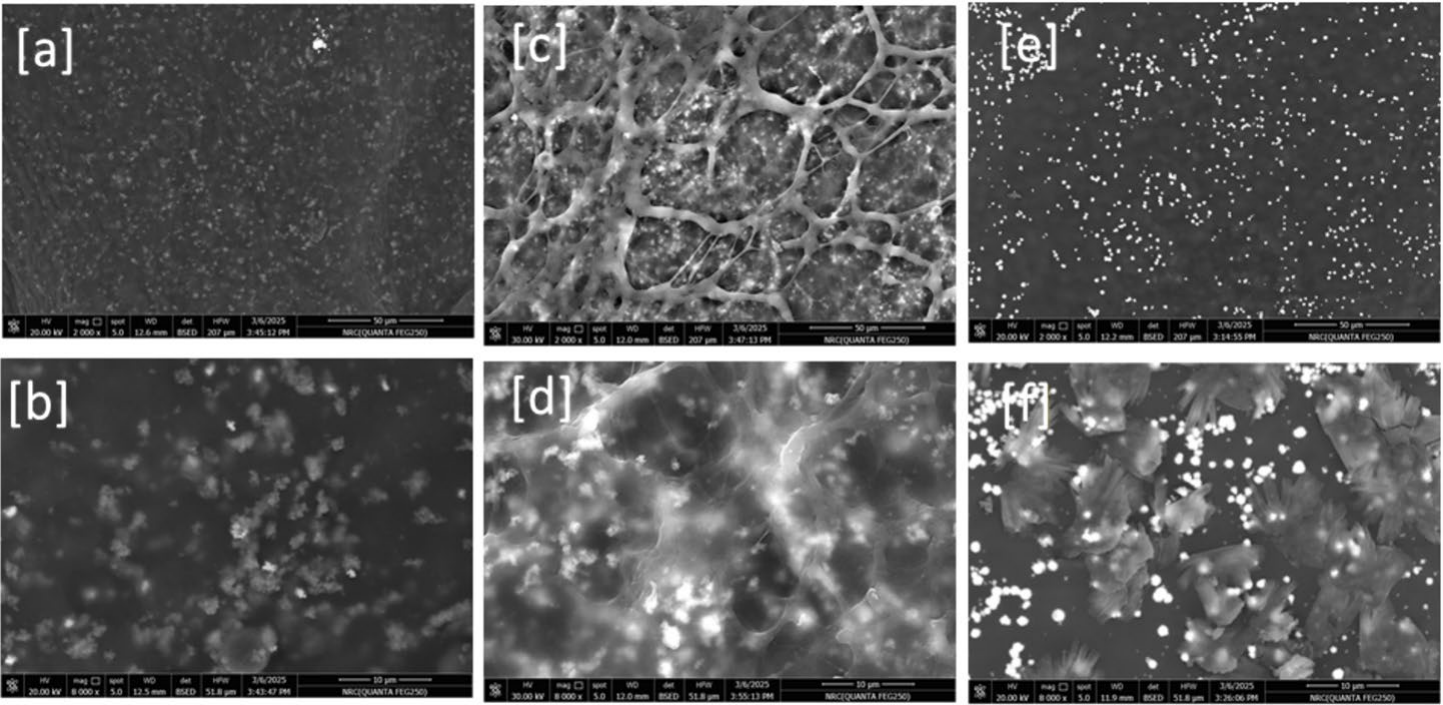
FE-SEM for the formulated nanocomposites: CR-1 with low (a) and high (b) magnifications, CR-2 with low (c)and high (d) magnifications, and CR-3 with low (e)and high (f) magnifications

The morphological structure of the nanocomposites is presented in Fig. 6. The formulation of the nanocomposite (1% extract) resulted in a film-like surface with a smooth morphology and small, grey particles of heterogeneous shapes (Fig. 6a). A high-magnification image revealed the particles as more aggregated and irregular in shape (Fig. 6b). The nanocomposite with a 3% extract exhibited a polymeric network that included the nanoparticles (Fig. 6c). The high-magnification image (Fig. 6d) was illustrative of this network acting as a scaffold for the particles to settle on. The formulated nanocomposite (5% extract) exhibited a surface morphology similar to that of various examined samples, with particles of more clearly defined shapes, such as spherical, less aggregated particles (Fig. 6e). Additionally, Fig. 6f clarifies the particles in spheres with a surface constructed from blocks related to the interaction between chitosan and collagen. The FE-SEM study illustrated the significant role of the curcuminoids-rich extract ratio in reducing and capping nanoparticles formed from metal salts, as well as utilizing polysaccharides as a template to stabilize the formulated nanocomposite. In conclusion, the ratio of curcuminoid-rich extract plays a limited role in the formulation of nanocomposites, enhancing their stability by up to 3%, which is in agreement with the DLS measurement results.

**Fig. 7:**
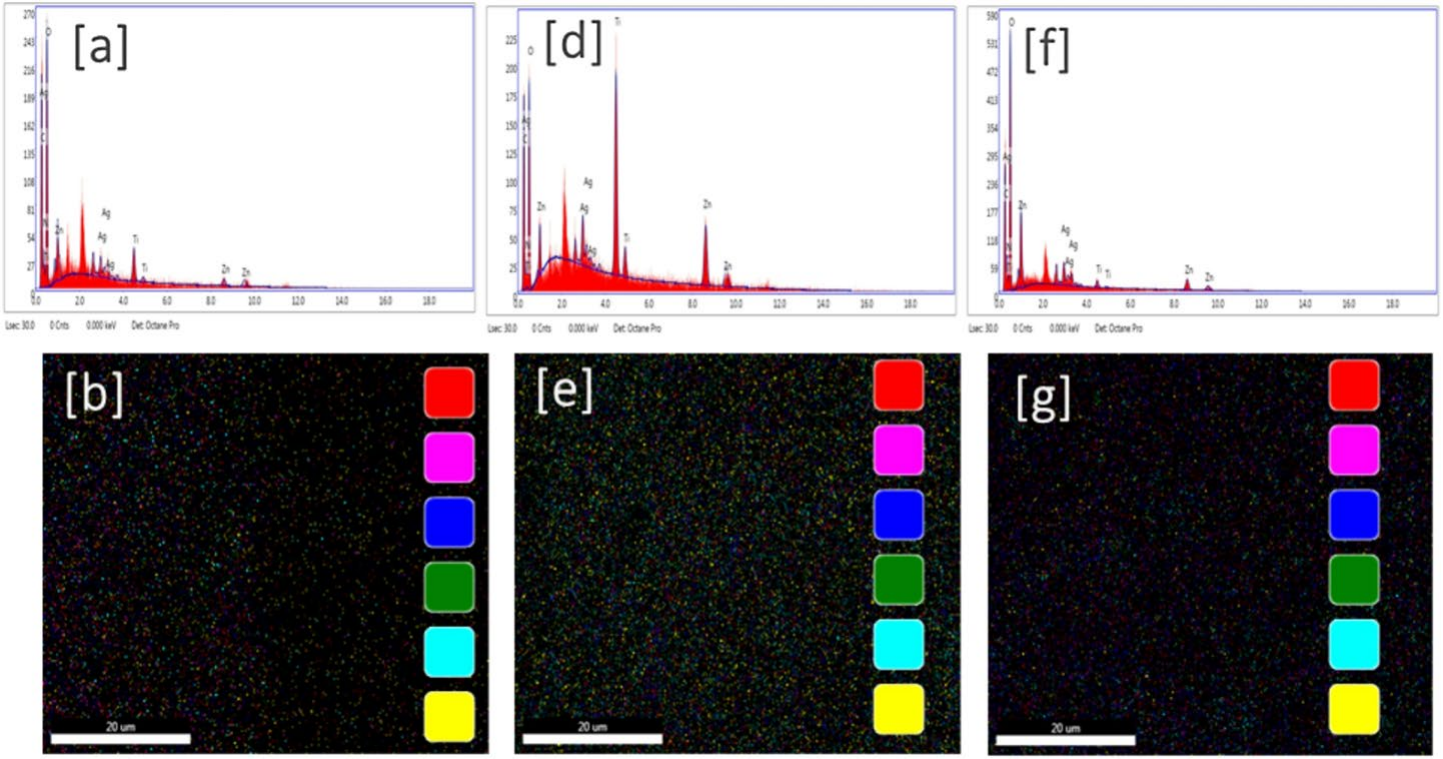
EDX and mapping of the formulated nanocomposites: CR-1 (**a**) and (b), CR-2 (**c**) and (d), and CR-3 (**e**) and (**f**), respectively

The EDX study with mapping is illustrated in Fig. 7. All formulated nanocomposites, using curcumin extract with ratios of 1%, 3%, and 5% (Fig. 7a, d, and f), respectively, according to their chemical composition, contained carbon, nitrogen, oxygen, silver, titanium, and zinc. A mapping study was presented on the uniform distribution of metal ions, which increases in parallel with the ratio of curcuminoids-rich extract, as observed in Figs. 7b, e, and **g**, respectively. Moreover, the sample CR-2 exhibited the highest contrast of metals compared to other nanocomposites. These results affirmed the conclusive observations from DLS measurements and FE-SEM studies as well.

**Fig. 8:**
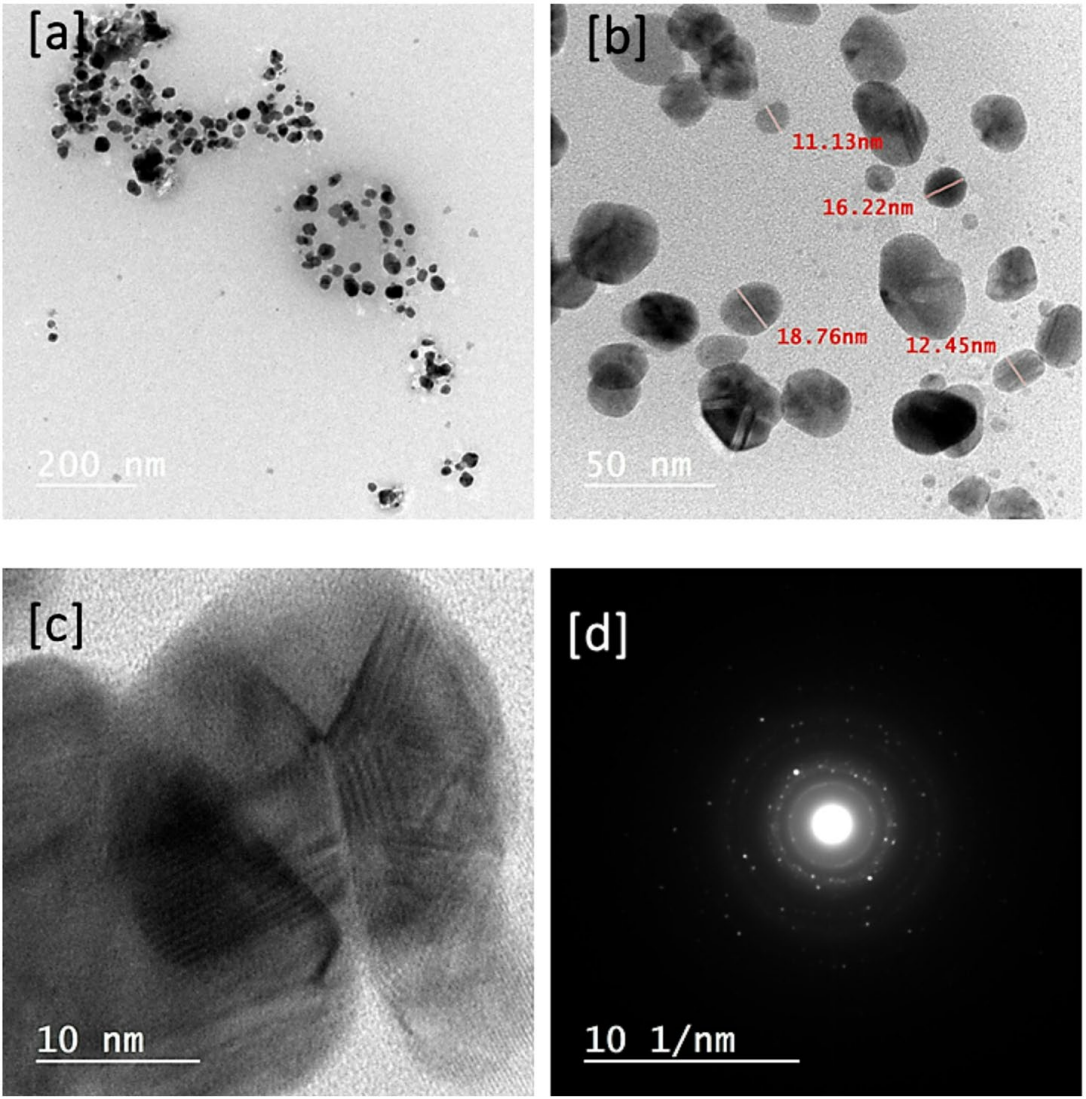
HR-TEM for the formulated nanocomposites; CR-2 with different magnifications from low to high, as a, b, and c, respectively, as well as the SAED (d)

HR-TEM images and SAED for the formulated nanocomposite with the highest ratio of curcuminoid-rich extract are shown in Fig. 8. Nanoparticles were observed as a group that was collected due to the role of the polysaccharide matrix. The particles were observed to be uniform in shape and varied in size (Fig. 8a). A magnified image is shown in Fig. 8b, illustrating particles of different sizes, ranging from 52 to 11 nm. The high-resolution image in Fig. 8c illustrates the lattice fringes of the nanoparticles, indicating interstitial incorporation due to the difference in d-spacing [26]. Otherwise, the SAED (Fig. 8d) illustrated that the bright spots signify scattered electrons that have constructively interacted, producing a diffraction pattern. These patterns represent a projection of the materials reciprocal lattice. The precise placement and intensity of the spots yield insights about the polycrystalline structure, orientation, and flaws of the material as well [59, 60]. ZnO presented the patterns 100, 002, and 101 [61]. TiO_2_ presents the patterns 101, 004, and 200 [62]. Ag presented the patterns 200 111, 220 [63].

### Biological Evaluation Results

#### Cell Viability Results

**Fig. 9:**
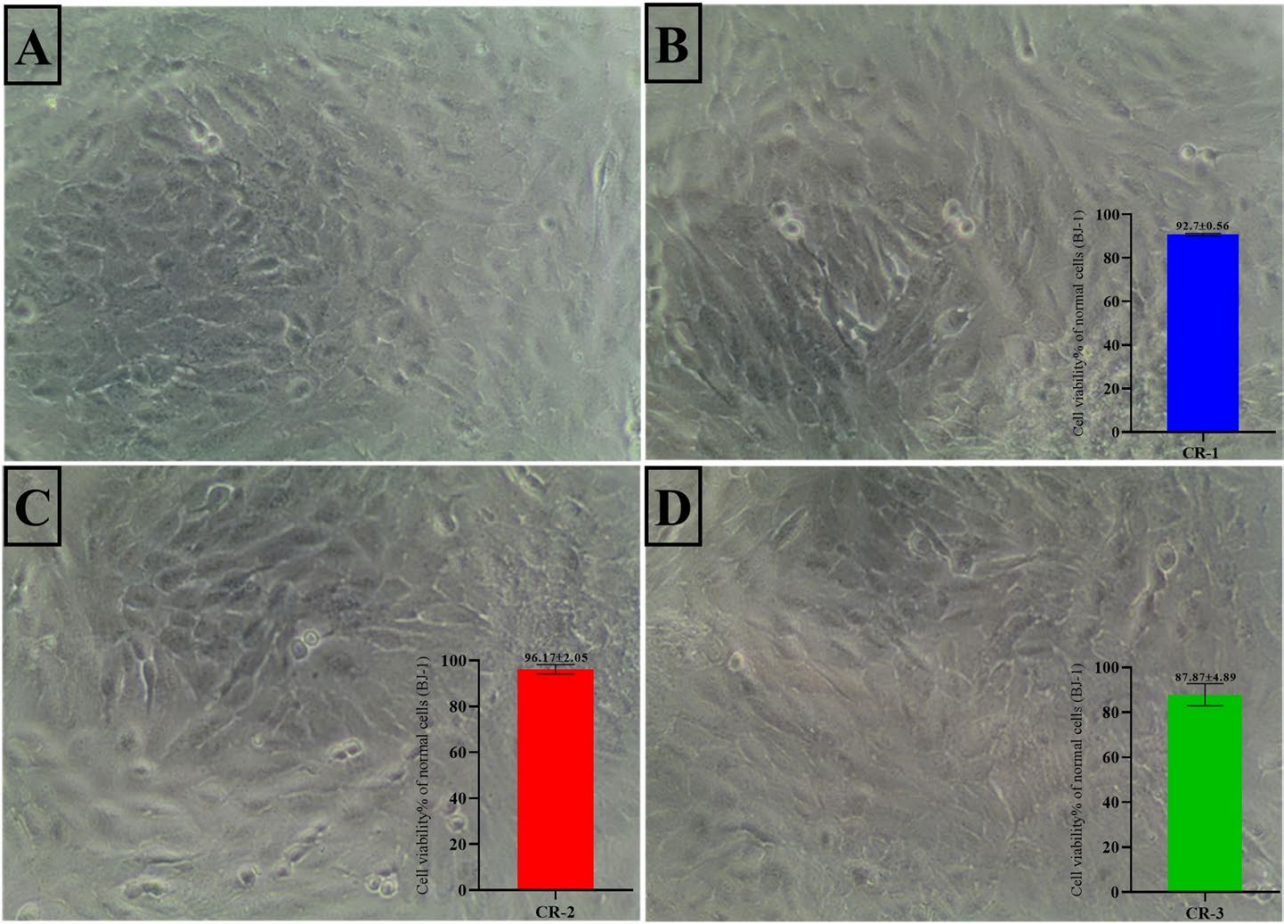
Cell viability % against skin normal fibroblast (BJ-1) of three Curcuminoids-rich extract formulas B (CR-1), C (CR-2), & D (CR-3) against untreated cells A. (Magnification 10x, Scale bar 200 μm)

The cytocompatibility of three formulated nanocomposites, CR-1, CR-2, and CR-3, was evaluated by assessing cell viability in normal human skin fibroblast cells (BJ-1) using an MTT assay (Fig. 9). Untreated cells (sample A) served as the negative control, and all tested samples were applied at a concentration of 100 µg/mL. Microscopic analysis was performed at 10x magnification with a 200 μm scale bar. The results revealed that the cell viability percentages for the samples were 90.7 ± 0.56% for CR-1, 96.17 ± 2.05% for CR-2, and 87.87 ± 4.89% for CR-3 when compared to the control. These findings suggest that all three curcumin formulations exhibit high biocompatibility with normal skin fibroblasts, indicating their potential safety for topical or biomedical applications. Among them, CR-2 demonstrated the highest viability (96.17%), indicating minimal cytotoxic effects and a favourable interaction with normal cells. The slightly lower viability observed in CR-3 (87.87%) may be attributed to differences in nanoparticle size, curcumin concentration, or surface characteristics, which could influence cellular uptake or oxidative stress. Overall, the results reinforce the potential of these green synthesised curcuminoids-rich extract-nanocomposites, particularly CR-2, as safe candidates for therapeutic or cosmetic use on skin tissues.

#### Antimicrobial Activity

**Fig. 10:**
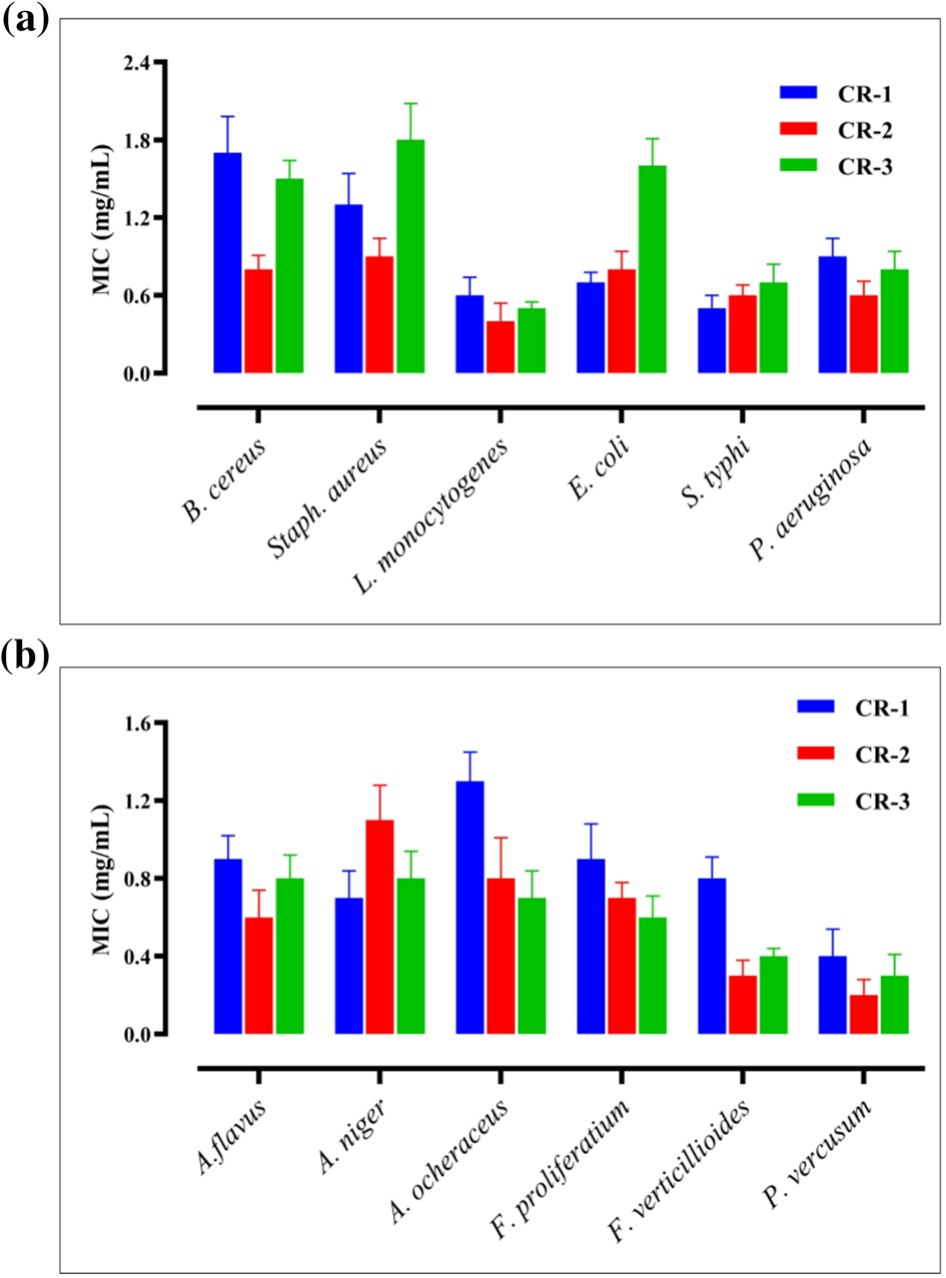
MIC for formula-based curcuminoids-rich extract against foodborne pathogenic bacterial strains (**a**). MIC for formula-based Curcuminoids-rich extract against mycotoxigenic pathogenic fungi strains (**b**).

Trimetallic nanoparticles (Ag@TiO₂@ZnO) were synthesized using varying concentrations of curcuminoids-rich extract (1%, 3%, and 5% *w/w*) as a green reducing and stabilizing agent. Among the prepared formulations, the CR-2 formula, presumably corresponding to the 3% curcuminoids-rich extract concentration, demonstrated promising antimicrobial activity. The antibacterial efficacy of CR-2 was evaluated against six pathogenic bacterial strains. The inhibition zones (mean ± SE, in mm) observed were as follows (Table 4): *L. monocytogenes* showed the highest susceptibility with a zone of 13.3 ± 1.25 mm, followed by *S. typhi* (11.5 ± 0.86 mm), *P. aeruginosa* (10.2 ± 0.76 mm), *B. cereus* (9.0 ± 0.50 mm), *S. aureus* (8.3 ± 0.58 mm), and *E. coli* (8.2 ± 0.28 mm) with MIC of 0.4 ± 0.14, 0.6 ± 0.08, 0.6 ± 0.11, 0.8 ± 0.11, 0.9 ± 0.14, and 0.8 ± 0.14 mg/mL, respectively (Fig. 10a). These results indicate that the nanoparticles exhibit broad-spectrum antibacterial activity, with more pronounced effects against Gram-positive bacteria and foodborne pathogens, such as *L. monocytogenes* and *S. typhi*. The nanoparticles showed broad-spectrum antibacterial activity, effective against both Gram-positive (e.g., *B. cereus*, *Staph. aureus*) and Gram-negative (e.g., *E. coli*, *P. aeruginosa*) bacteria. The highest activity was observed against *L. monocytogenes*, indicating high susceptibility, which may be due to differences in membrane permeability or metabolic response. The moderate activity observed against *P. aeruginosa* and *S. typhi* reflects the potential of the nanoparticle in targeting enteric and opportunistic pathogens. In addition, the activity against *E. coli* and *S. aureus* was comparatively lower, suggesting strain-specific resistance or lower diffusion in the media.

**Table 4:** Antibacterial activity of formulated nanocomposites

Bacterial strains	Inhibition Zone mm (Mean ± S.E)				
	Negative control	Positive control	CR-1	CR-2	CR-3
*B. cereus*	0	8.7 ± 0.58	7.5 ± 0.50	9.0 ± 0.50	7.8 ± 1.04
*Staph. aureus*	0	14.0 ± 1.32	8.0 ± 0.86	8.3 ± 0.58	7.2 ± 0.28
*L. monocytogenes*	0	34.0 ± 0.76	13.3 ± 0.54	13.3 ± 1.25	12.5 ± 1.80
*E. coli*	0	23.0 ± 0.78	9.0 ± 0.50	8.2 ± 0.28	7.7 ± 0.15
*S. typhi*	0	27.7 ± 1.04	12.8 ± 0.84	11.5 ± 0.86	10.3 ± 0.76
*P. aeruginosa*	0	15.5 ± 1.78	8.2 ± 0.28	10.2 ± 0.76	8.0 ± 0.50

*n* = 3, *S.E: standard error,* 0: No inhibition*,* Negative control: DMSO*,* Positive control: tetracycline (. CR-00: extract only*,* CR-1*,* 2*,* 3: the three preparations*

The CR-2 formulation was tested against six common and mycotoxigenic fungal strains (Table 5). The inhibition zones recorded were 13.2 ± 0.76 mm for *P. verrucosum* (with MIC of 0.2 ± 0.08 mg/mL), 11.8 ± 0.76 mm for *F. verticillioides* with MIC of 0.3 ± 0.08 mg/mL, 9.3 ± 1.04 mm for *Aspergillus ochraceus* with MIC of 0.8 ± 0.21 mg/mL, 9.2 ± 0.28 mm for *F. proliferatum* with MIC of 0.7 ± 0.08 mg/m.L, 8.8 ± 0.76 mm for *A. flavus* with MIC of 0.6 ± 0.14 mg/mL, and 8.2 ± 1.25 mm for *A. niger* with MIC of 1.1 ± 0.18 mg/mL (Fig. 10b). The results reveal notable antifungal potential, particularly against toxigenic strains such as *P. verrucosum* and *F. verticillioides*, suggesting potential applications in food safety, agriculture, and biomedical fields.

**Table 5:** Antifungal activity of formula-based curcuminoid-rich extract

	Inhibition Zone mm (Mean ± S.E)				
Fungi strains	Negative control	Positive control	CR-1	CR-2	CR-3
A. *flavus*	0	26.0 ± 0.50	8.7 ± 1.04	8.8 ± 0.76	9.5 ± 1.32
*A. niger*	0	24.5 ± 0.76	9.5 ± 1.04	8.2 ± 1.25	8.5 ± 0.50
*A. ocheraceus*	0	23.2 ± 0.84	8.2 ± 0.15	9.3 ± 1.04	8.2 ± 0.28
*F. proliferatium*	0	28.0 ± 0.50	8.0 ± 0.50	9.2 ± 0.28	8.8 ± 0.28
*F. verticillioides*	0	25.8 ± 0.76	9.5 ± 0.18	11.8 ± 0.76	10.3 ± 1.25
*P. verrcusum*	0	28.5 ± 0.50	10.2 ± 1.61	13.2 ± 0.76	11.8 ± 0.76

*n* = 3, *S.E.: standard error, 0: No inhibition, Negative control: DMSO, Positive control: Miconazole.

The formulation demonstrated noteworthy antifungal activity, particularly against mycotoxigenic fungi such as *P. verrucosum* and *F. verticillioides*. *P. verrucosum* was the most affected organism towards treatments, showing strong potential for food preservation and agricultural applications. The lowest affected microorganisms were *A. ocheraceus* and *A. niger*, which may indicate partial resistance or structural limitations in nanoparticle-fungal interactions. Overall, the CR-2 formula, based on intermediate curcumin concentration, demonstrated balanced and effective antimicrobial activity. The observed bioactivity may be attributed to the synergistic effects of trimetallic nanoparticles, silver, titanium dioxide, and zinc oxide, further enhanced by the biological functionality of a curcumin-rich extract. These findings support the potential of green-synthesised trimetallic nanoparticles in combating a wide range of microbial pathogens [64, 65]. Turmeric (*Curcuma longa*) has been traditionally recognized for its medicinal properties, primarily attributed to its active compound, curcumin. Numerous studies have investigated the antimicrobial properties of turmeric extract, demonstrating its potential against various pathogens, including bacteria, fungi, and viruses. A significant body of research has focused on the antibacterial effects of turmeric extract. Recent studies have demonstrated that curcumin exhibits varying degrees of antimicrobial activity against both Gram-positive and Gram-negative bacteria. For instance, *Uthman*,* A.I.*,* et al. 2024* [66] reported that curcumin inhibited the growth of *S. aureus* and *E. coli*, with MICs of 25 mg/mL and 100 mg/mL, respectively. Mechanistically, curcumin disrupts bacterial cell membranes and inhibits essential metabolic pathways, leading to cell death [67]. Moreover, turmeric extract has also shown promise in combating fungal infections. Studies have highlighted its effectiveness against various fungi, including *C. albicans* and *A. niger*. *Zhang*,* D.*,* et al. 2023* [68] illustrated that turmeric extract demonstrated antifungal properties by impairing fungal cell wall synthesis and inducing apoptosis in fungal cells. Continued research into formulations that enhance bioavailability and effectiveness will be crucial in translating these findings into clinical practice [65]. In this study, the formulated nanocomposites offer an eco-friendly antimicrobial agent against a broad spectrum of pathogens, including some dangerous food-borne pathogenic bacteria and fungi, with a safe profile, making them a promising candidate for future food and health applications.

The antimicrobial properties of nanomaterials can be explained through multiple and interconnected mechanisms, including cell wall disruption, generation of oxidative stress, and interference with intracellular components. Trimetallic nanoparticles, such as Ag, TiO₂, and ZnO, can attach to microbial cell walls, causing a loss of permeability and leakage of cellular contents. They also generate reactive oxygen species (ROS) that damage proteins, lipids, and DNA, while released metal ions interact with enzymes and genetic material, inhibiting vital metabolic and replication processes. Similar mechanisms occur against fungi, leading to the inhibition of spore germination, hyphal damage, and reduced mycotoxin production [69, 70].

Also, the antimicrobial and cytocompatibility results of the CR-2 nanocomposite can be directly related to the structure–activity relationship (SAR) of curcuminoids. In which the presence of curcumin and demethoxycurcumin, which contain one or/two methoxy groups on the aromatic rings. These methoxy groups increase lipophilicity, enhancing membrane penetration, ROS generation, and interaction with microbial proteins and DNA, thereby boosting antimicrobial potency. In contrast, bisdemethoxycurcumin, which lacks methoxy groups, contributes less to direct antimicrobial activity due to its higher polarity and lower membrane affinity. However, all curcuminoid components maintain high biocompatibility, as reflected in the negligible cytotoxicity of CR-2 (~ 3.83% at 100 µg/mL) toward human skin fibroblasts. Thus, the combination of curcuminoids with different methoxy substitution patterns in the CR-2 nanocomposite enables synergistic antimicrobial activity while preserving safety, making it a promising and safe candidate. Overall, these mechanisms underscore the robust antibacterial and antifungal properties of nanomaterials, rendering them promising candidates for applications in agriculture, food safety, and biomedical fields.

## Conclusion

This study successfully developed trimetallic nanocomposites (Ag@TiO₂@ZnO) *via* a green synthesis approach, utilizing a curcuminoids-rich extract as a natural reducing and capping agent. Chitosan and collagen were used to enhance the biocompatibility and stability of the formulation. Among the tested formulations, CR-2, based on a 3% (w/w) curcuminoids-rich extract, emerged as the most effective, exhibiting strong antimicrobial activity and excellent cytocompatibility with human skin fibroblasts (BJ-1), with a high cell viability of 96.17% (low toxicity at a high concentration of 100 µg/mL). The CR-2 formula demonstrated significant antibacterial activity against a range of foodborne pathogens, notably *L. monocytogenes*, *S. typhi*, and *P. aeruginosa*, as well as potent antifungal effects against mycotoxigenic fungi such as *P. verrucosum* and *F. verticillioides*. These findings are particularly relevant for food safety, as contamination with such pathogens poses a serious threat to public health and food security. The ability of CR-2 to inhibit both bacterial and fungal pathogens underscores its potential as an effective antimicrobial agent in food packaging, preservation systems, and surface sanitization. The integration of natural bioactive components, such as curcuminoid-rich extracts, together with metal oxide nanoparticles in a chitosan-collagen matrix provides a biocompatible, eco-friendly, and sustainable platform. This aligns with global efforts to substitute synthetic chemical preservatives in food and shift toward safer, greener alternatives. Overall, the CR-2 formulation stands out as a multifunctional nanocomposite with promising applications in the food and biomedical sectors, contributing to the development of next-generation antimicrobial technologies that combine efficacy with minimal cytotoxic impact. In the future, further studies should be conducted to evaluate its effects on non-target organisms and in vivo models, thereby confirming and reinforcing its biocompatibility profile.

## Data Availability

The datasets used and/or analyzed during the current study are available from the corresponding author upon reasonable request.
